# Integrating Single-Cell and Bulk RNA Sequencing Data to Explore Sphingolipid Metabolism Molecular Signatures in Ovarian Cancer Prognosis: an Original Study

**DOI:** 10.7150/ijms.107391

**Published:** 2025-03-24

**Authors:** Xu Huang, Xiaoyu Li, Wulin Shan, Yingyu Dou, Qiongli Yu, Yao Chen, Zengying Wang, Haomin Zhang, Yumeng Wang, Xiaofei Lu, Wenju Peng, Bairong Xia

**Affiliations:** 1 The First Affiliated Hospital of USTC, Division of Life Sciences and Medicine, University of Science and Technology of China, Hefei, Anhui, 230031, China.; 2 Bengbu Medical University, Bengbu, Anhui, 233000, China.; 3 Department of Obstetrics and Gynecology, The First Affiliated Hospital of USTC, Division of Life Sciences and Medicine, University of Science and Technology of China, Anhui Provincial Cancer Hospital, Hefei, Anhui, 230031, China.

**Keywords:** Ovarian cancer, Sphingolipid metabolism, Immune infiltration, Chemotherapy drug sensitivity, GBP5

## Abstract

**Background:** Ovarian cancer (OC) is the deadliest malignant tumor in the female reproductive system. Sphingolipid metabolism (SM) is crucial for cellular function and has been linked to OC progression. Dysregulation of sphingolipid pathways contributes to tumor growth, chemoresistance, and metastasis in OC. Currently, investigations into the relationship between sphingolipid-related genes (SRGs) and OC prognosis in their initial stages. Our study aimed to develop a novel molecular subtyping based on SRGs and construct a signature to predict the prognosis of patients with OC, immune cell infiltration characteristics, and chemotherapy sensitivity.

**Methods:** Bulk and single-cell RNA-sequencing data of OC was analyzed primarily from the TCGA and GEO databases. The gene set related to the sphingolipid pathway (hsa00600) was selected from the SM pathway, and the enrichment of SRGs was analyzed in the annotated single-cell sequencing data. The Scanpy function was used to score the gene features of each cell and further identify differentially expressed genes. By intersecting with the genes most closely related to SM activity identified through Weighted Gene Co-expression Network Analysis (WGCNA) based on bulk RNA sequencing data, and after performing univariate COX, multivariate COX and LASSO regression, three SRGs were identified. Subsequently, the SRGs-related prognostic signature was constructed. The analysis was further extended to clinical feature correlation, GSEA, tumor microenvironment (TME) analysis and chemotherapy sensitivity analysis. Finally, the expression and function of the key gene GBP5 in the model were validated through *in vitro* experiments.

**Results:** Compared to other sites, SRG scores were highest in ascites, and among different cell types, SRG scores were highest in T cells. By integrating scRNA-seq and bulk RNA-seq analysis, three SRGs (C5AR1, GBP5, and MARCHF3) were ultimately selected to develop a prognostic model for SRGs. In this model, patients with higher risk scores had shorter overall survival, which was validated in the testing cohort. Immune infiltration analysis revealed that the risk score was negatively correlated with the abundance of CD8+ T cell infiltration and positively correlated with the abundance of M2 macrophage infiltration. Chemotherapy sensitivity analysis showed that the high-risk group exhibited increased resistance to Oxaliplatin, Gemcitabine, and Sorafenib. *In vitro*, we demonstrated that knockdown of the protective gene GBP5 in HEYA8 and SKOV3 cells enhanced cell viability, proliferation, and invasiveness, reduced apoptosis, and increased IC50 values for chemotherapy drugs.

**Conclusion:** Our model effectively identifies high-risk patients and provides a reference for prognosis prediction using SRG signature. Moreover, hub gene GBP5 acts as a tumor inhibitory factor and regulates the chemosensitivity of oxaliplatin, gemcitabine, and sorafenib in OC.

## Introduction

Ovarian cancer (OC) remains the most lethal gynaecological malignancy with 314 000 cases and 207 000 deaths annually worldwide 1. Epithelial OC (EOC), comprising 90% of cases, is often diagnosed at advanced stages due to nonspecific early symptoms, leading to widespread chemoresistance in recurrent disease 2. While molecular therapies and immunotherapy have improved outcomes for some advanced EOC patients 3, treatment efficacy remains limited by tumor heterogeneity and an immunosuppressive microenvironment 4. Consequently, this underscores the urgent need for biomarkers that stratify patients by risk and guide personalized therapies.

Sphingolipids are biologically active lipids with a sphingoid backbone that maintain the barrier function and fluidity of cell membranes 5. Sphingolipid metabolism (SM) is increasingly recognised as a master regulator of oncogenic processes. Central to its function is the enzymatic regulation of the ceramide (Cer)-sphingosine-1-phosphate (S1P) rheostat — a biochemical switch governing cellular apoptosis versus survival 6,7. This balance is profoundly disrupted in ovarian malignancies, where SM dysregulation has been mechanistically linked to ascites-driven metastasis and platinum resistance through PD-L1-enriched extracellular vesicle signalling 13,14. Elevated Cer/S1P ratios in patient ascites and plasma are strongly associated with advanced disease 9,14, whilst longitudinal cohort data further suggest that elevated levels of circulating SMs 3 to 23 years prior to diagnosis were associated with an increased risk of ovarian cancer, with the correlation being particularly pronounced in postmenopausal women 9. Despite mounting evidence implicating sphingolipid dysregulation in ovarian cancer progression, the clinical translation of sphingolipid-related gene (SRG) signatures remains hindered by two unresolved questions: first, the uncharacterised prognostic value of SRG profiles in patient stratification, and second, the poorly defined mechanistic interplay between SRG-driven tumour microenvironment remodelling and emergent chemoresistance phenotypes.

In this study, we hypothesize that SRGs-based molecular subtyping can predict OC outcomes and uncover TME-driven therapeutic vulnerabilities. To address these gaps, we integrated single-cell and bulk RNA sequencing from TCGA and GEO databases to: (i) Identify SRGs driving SM pathway dysregulation in OC; (ii) Construct a prognostic signature validated across independent cohorts; (iii) Conduct drug sensitivity analyses and examined changes in immune infiltration through cluster analysis; (iv) Functionally validate key SRGs *in vitro*. This study pioneers a systematic framework to bridge SM biology with clinical translation in OC management.

To sum up, our findings establish the first multi-omics-derived SRGs framework for OC risk stratification and therapy personalization.

## Methods

### Bulk RNAseq data collection

To explore the fundamental molecular drivers of chemoresistance in ovarian cancer, we gathered bulk RNA-seq data alongside clinical profiles of high-grade serous ovarian cancer (HGSOC) patients sourced from TCGA (https://portal.gdc.cancer.gov/). Patients lacking essential clinical pathological details or overall survival (OS) data were systematically excluded from our study. Employing the 'limma' package effectively minimized potential batch effects. Additionally, for robust external validation, matrix files from GSE26712 (n=185, Platform = GPL96), GSE32062 (n = 260, Platform = GPL6480), and GSE14764 (n=80, Platform = GPL96) were obtained from the GEO database (https://www.ncbi.nlm.nih.gov/geo/), providing diverse and independent datasets enriched with comprehensive clinical and survival metrics.

### Single-cell RNAseq data acquired and processing

The single-cell dataset GSE180661 of OC was retrieved from the CELLXGENE database, comprising 156 samples collected from 41 patients diagnosed with HGSOC. These samples originated from multi-site tissues obtained during pre-treatment laparoscopy or primary debulking surgeries. Specifically, the dataset includes untreated samples encompassing primary adnexal tumors (ovary and fallopian tube), metastatic sites (omentum, bowel, pelvic peritoneum, upper quadrants), and ascites. Quality control of the single-cell RNA sequencing (scRNA-seq) data was conducted using the 'scanpy' Python package, resulting in a normalized and scaled gene-by-cell matrix for each sample. Cells included for subsequent analysis exhibited expression of at least 500 genes and 1,000 unique molecular identifier (UMI) counts, while maintaining mitochondrial gene expression below 25%. Cell cycle phase assignment utilized the Seurat CellCycleScoring function, with Scrublet (version 0.2.1) employed to identify and exclude cells with a doublet score exceeding 0.25. Patient-level sample matrices were merged and subsequently re-normalized and scaled using default Seurat functions, resulting in a total of 929,690 eligible cells for further investigation. Principal component analysis (PCA) was applied to the filtered feature-by-barcode matrix, with uniform manifold approximation and projection (UMAP) embeddings generated based on the first 50 principal components, encompassing cohort-level and patient-level embeddings for major cell types. UMAP embeddings of major cell type supersets were constructed based on 50 batch-corrected harmony components, with six distinct supersets identified: 1 T cells; 2 B cells and plasma cells; 3 myeloid cells, dendritic cells (DCs), and mast cells; 4 fibroblasts; 5 endothelial cells; and 6 ovarian cancer cells. Batch correction using the R package harmony (version 0.1) was applied to each superset to mitigate patient-specific effects, ensuring robust downstream analyses.

### The acquisition of sphingolipid-related genes

The gene set related to the sphingolipid pathway was curated from the Sphingolipid metabolism pathway (hsa00600), based on the KEGG pathway database.

### Expression signature analysis

To determine the enrichment of sphingolipid-related genes in the single-cell dataset, we used the Scanpy function scanpy.tl.score_genes() to score the gene signature for each cell.

### Single sample gene set enrichment analysis

To calculate the precise score of a gene set enriched in a sample, the ssGSEA function of “gsva” R package was utilised. In this study, ssGSEA analysis was used to determine the SM scores for each TCGA-OV patient.

### Weighted co-expression network analysis

The 'WGCNA' package within the R environment was employed to execute the Weighted Gene Co-expression Network Analysis (WGCNA), a systems biology methodology utilized for the construction of the TCGA-OV gene co-expression network. Leveraging the interconnectedness among gene sets and their correlation with phenotypic attributes, WGCNA facilitated the detection of gene sets exhibiting high covariance and offers insights into potential biomarker genes or therapeutic targets. This study utilized WGCNA to delineate gene modules linked to the SRGs score in OC and to pinpoint the corresponding associated genes.

### Risk signature associated with sphingolipid metabolism

Initially, a univariate Cox analysis was employed to identify sphingolipid-related genes (SRGs) with prognostic significance. Subsequently, a prognostic model was constructed through Lasso regression to refine the selection of prognostic SRGs. Subsequent to model construction, each ovarian cancer case was assigned a risk score utilizing the developed algorithm. Utilizing the median risk score as a threshold, patients within the TCGA-OV cohort were stratified into high-risk and low-risk categories. Subsequent, to evaluate the impact of clinical features and risk scores on prognosis, multivariate analysis (including race, age, and pathological stage) was conducted for each risk group. Then, the results were represented and quantified using a column chart, which calculated the survival probabilities of OC patients at 1, 3, and 5 years. Use decision curve analysis (DCA) to determine overall clinical benefits, which compares the net benefits provided by risk scores and clinical features.

### Immune infiltration analysis

We conducted an investigation into the correlation between prognostic models and tumor immunity alongside immunotherapy. The extent of immune infiltration among OC patients within the TCGA database was ascertained utilizing the TIMER 2.0 database, which encompasses outcomes from seven distinct evaluation methodologies. Heatmaps were subsequently generated to depict the relative proportions of immune cell infiltration within the tumor microenvironment (TME) based on these data. Following this, single-sample Gene Set Enrichment Analysis (ssGSEA) was performed on genes within the prognostic risk assessment model utilizing the R package GSEABase, focusing on immune-related properties. Utilizing the "estimate" R package, users were able to discern the relative abundance of stromal cells, immune cells, and tumor cells, thereby facilitating comparisons across different risk strata.

### Mutation landscape and drug sensitivity analysis

The "maftools" software was employed to derive the genetic mutation profiles of OC patients sourced from the TCGA database. Subsequently, the comprehensive genetic mutation datasets were integrated with the risk score. Moreover, the half-maximal inhibitory concentrations (IC50) of prevalent chemotherapeutic agents were computed utilizing the calcPhenotype function of "oncoPredict" R package enabling the investigation of the association between the risk score and drug responsiveness. The IC50 values were juxtaposed between the distinct risk cohorts through Wilcoxon signed-rank tests.

### Online database analysis

The association between GBP5 gene expression and overall survival (OS) and staging of ovarian cancer was analyzed using the Gene Expression Profiting Interactive Analysis (GEPIA) database, which includes TCGA and GTEx samples 13.

### Cells and cell culture

A human normal ovarian epithelial cell line (IOSE-80) and ovarian cancer cell lines (A2780, OVCAR8, SKOV3, HEYA8 and CAOV3) were purchased from EK-Bioscience (Shanghai, China). These cell lines are all derived from epithelial tissue and have been studied within the sphingolipid metabolism pathway Table S3. A2780 OVCAR8 cells were maintained in RPMI 1640 (Hyclone, Cytiva, UT, USA) supplemented with 10% fetal bovine serum (FBS) at 5% CO2 at 37°C. SKOV3 was cultured in McCoy's 5a (Hyclone, Cytiva, UT, USA) medium and 10% FBS. HEYA8 and CAOV3 were cultured in DMEM medium (Hyclone, Cytiva, UT, USA) and 10% FBS. OVCAR3 were cultured in 1640 medium (Hyclone, Cytiva, UT, USA), 10% FBS and 0.01mg/ml insulin (Beyotime, Shanghai, China).

### Immunohistochemistry (IHC)

Tissue samples from 10 cases of early-stage (I-II) and late-stage (III-IV) ovarian tumours were collected from the Gynaecologic Oncology Department of the First Affiliated Hospital of the University of Science and Technology of China (West District). The tumour samples were classified as high-grade serous ovarian cancer by experienced pathologists and staged by gynaecologic oncology specialists. None of the patients had received any treatment prior to surgery and all signed informed consent forms. All tissues were frozen in liquid nitrogen within 30 minutes after resection and stored at -80 °C. Tumor tissues were fixed in 10% formalin, embedded in paraffin, and sectioned into 4-6 µM sections. After deparaffinization, rehydration, and microwave antigen retrieval, slides were incubated with GBP5 antibody (1:500, Proteintech, China). This study was approved by the First Affiliated Hospital of the University of Science and Technology of China (West District) (2023-FLK-01).

### Reverse transcription quantitative PCR assay (RT-qPCR)

Total RNA was extracted from cells or plasma using TRIZOL reagent (Invitrogen, Waltham, MA, USA). The RNA was reverse transcribed into cDNA using a PrimeScript RT Reagent Kit (Beyotime, Shanghai, China). qPCR was performed using SYBR Green qPCR Master Mix (Beyotime, Shanghai, China) on a ftc-3000p PCR instrument (fungyn biotechinc, Canada). cDNA was synthesized from total RNA (500ng) at 48°C for 30 min and 95°C for 10 min. cDNA (1 μg) was subjected to polymerase chain reaction (PCR) for 45 cycles of 94°C for 45 seconds, 56°C for 30 seconds, and 72°C for 30 seconds. Primers were synthesized by RiboBio (Guangzhou, China). Primer sequences were as follows (5′→ 3′): GBP5 forward: CTGTCTGCCATTACGCAACCTG, GBP5 reverse: GTGTGAGACTGCACCGTAGATG, GAPDH forward: GAACGGGAAGCTCACTGG, GAPDH reverse: GCCTGCTTCACCACCTTCT.

### RNA interference

SiRNA against OLFML1 was provided by GeneAdv (Suzhou, China). Si-NC was used as a negative control. Transfection was performed with GA-RNA Transfection Kit (GeneAdv, China) according to the manufacturer's instructions. The siRNA sequences as follows:

### Western blot analysis

The proteins were quantified by the BCA Protein Assay Kit (Beyotime, Shanghai, China). The Western blotting was performed according to the standard protocol using GBP5 antibody (1:2000, Proteintech, China) and GAPDH antibody (1: 10000, Protintech, China) and blots was detected using the ECL assay kit (Thermo Scientific, Waltham, MA, USA).

### Cell counting kit‑8 (CCK‑8) assay

The Cell Counting Kit-8 (CCK-8; BestBio, Nanjing, China) was used to detect cell proliferation. We seeded the cells in 96-well plates at 2×10^3^ cells per well. The plate was then incubated with 10 ml CCK-8 labeling reagent (A311-01, Vazyme, Nanjing, China) per well for 2 hours in the dark at 37°C. The absorbance of the cells was measured at 450 nm wavelength with the enzyme-labeled meter (A33978, Thermo, USA) to analyse the viability of the cells. It was detected for 0, 24, 48, 72 and 96 hours. For drug sensitivity testing, the sensitivity of the above drugs was evaluated by treating oxaliplatin, gemcitabine, and sorafenib in a 96 well plate for 48 hours before adding CCK-8 solution.

### Colony formation

We transfected 1000 cells and kept them in 6-well plates for approximately 14 days. Two weeks later, we saw the cell clones with the naked eye. Next, the cells were rinsed and fixed for 15 minutes in 4% paraformaldehyde (Biosharp, China). Crystal violet (Beyotime, China) staining was performed for 20 minutes, dried at room temperature, and counted per well.

### Transwell assay

Migration and invasion assays were performed using Corning 24 well inserts with or without Matrigel (BD Biosciences, USA). 1×10^5^ cells without FBS were seeded into the upper part of the chamber while medium plus 10% FBS was loaded to the lower side. The migrated and invased cells were fixed, stained, and then counted after 24 h or 48 h. The cells were fixed with 4% paraformaldehyde (Biosharp, China), stained with 0.1% crystal violet (Beyotime, China), and counted under a light microscope.

### Wound healing assay

1.5×10^5^ cells were seeded in a 24 well plate. After the cells were cultured to 80-90% confluence, they were replaced with serum-free medium and incubated for 8 h. Scratches were made using a sterile pipette tip. The cell healing was regularly observed.

### Apoptosis analysis

To study apoptosis, an Annexin V-FITC/PI (MULTI SCIENCES, China) kit was used. After washing the tumor cells with PBS twice, 1×10^6^ cells were suspended in 500 μL 1× binding buffer and incubated in the dark with 5 μL Annexin V-FITC for 10 min then 10 μL PI was added for 5 min. Immediately measured by flow cytometry (LSRFortessa, BD Biosciences, USA), and 10,000 signals were collected from each sample. FlowJo software was used for further analysis.

### Statistical analysis

All analyses were executed through R (version 4.1.2), GraphPad Prism (version 8.0), and FlowJo (version 10.7.2). All experimental data were presented as means ± standard deviation (SD). P-value < 0.05 was considered significant.

## Results

### Research process and single-cell sequencing data analysis

**Figure 1:**
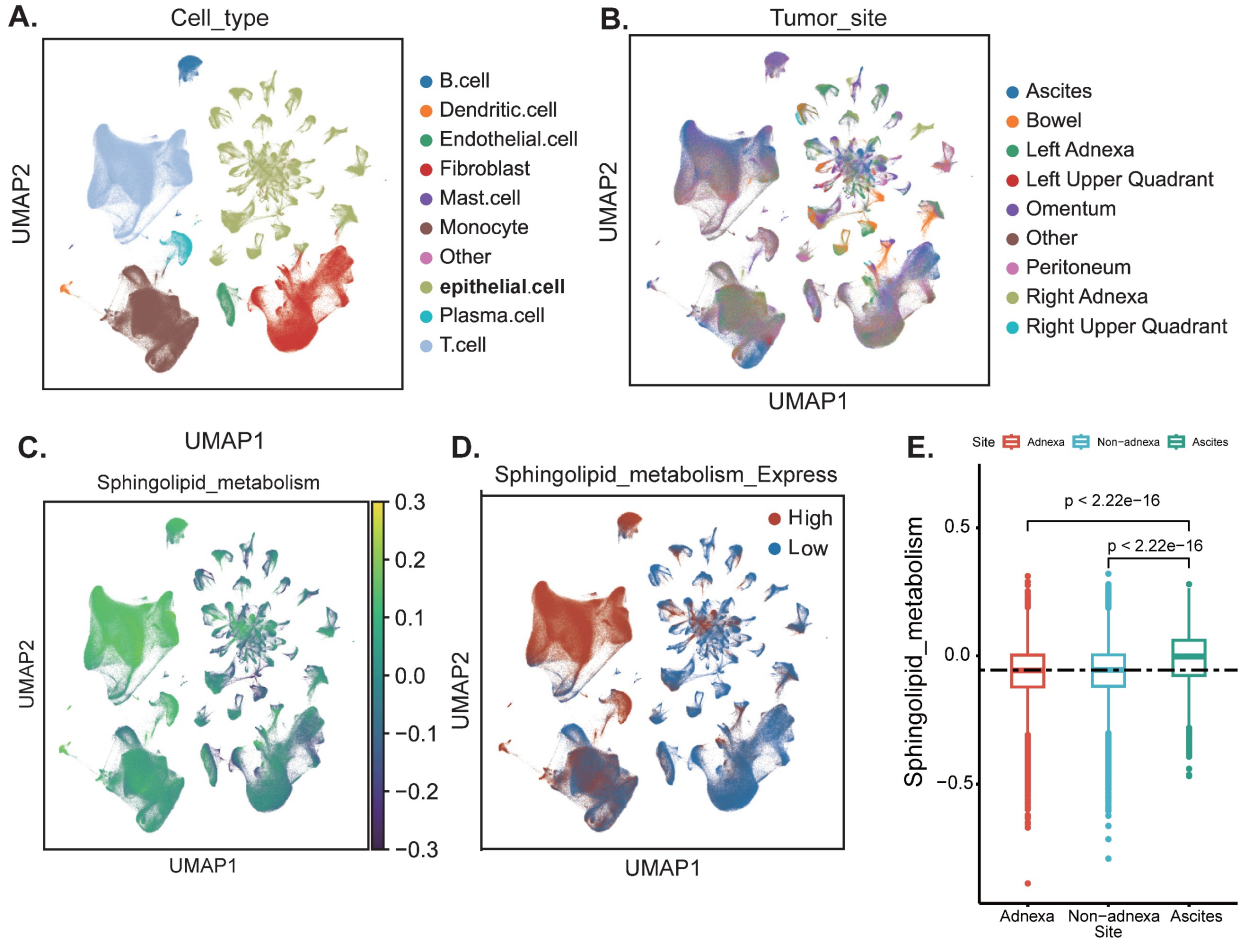
Annotation of cell subsets and identification of differentially expressed genes. (A) The results of the dimension reduction cluster analysis are shown in the UMAP diagram. Cells were annotated into 10 different types of cells. (B-D) All cells were scored according to sphingolipid-associated genes (SRGs) and were divided into high and low groups. (E) Analyzing the expression differences of genes related to sphingolipid metabolism in different tissue sources, the results showed that in ascites, genes related to sphingolipid metabolism were significantly higher than those in the attachment and non-attachment regions, *P*<2.2e-16.

The flowchart of this work is shown in graphical abstract. Firstly, our study centres on the enrichment of sphingolipid metabolism at the single-cell level. After rigorous quality control and sample integration, we retained multi-site tissue biopsy samples (n=160) from 42 patients with newly diagnosed OC. Cells retained for analysis had a minimum of 500 expressed genes and 1,000 UMI counts and had less than 25% mitochondrial gene expression, ensuring there were no discernible batch effects. Major cell type assignments were computed for each patient with CellAssign (version 0.99.2) using a set of curated marker genes 14. Drawing on the expression of cell type marker genes from prior research conducted by Vázquez-García *et al.*, we classified these clusters into ten distinct cell types, including B cells, dendritic cells, endothelial cells, fibroblasts, macrophages, monocytes, epithelial cells, stromal cells, T cells, and other cells (Figure 1A) 15. Subsequently, we illustrated that the anatomical sites of sample collection primarily originated from the adnexal region (encompassing both the left and right ovaries), non-adnexal regions (such as the omentum, peritoneum, intestine and other internal abdominal sites), and ascites (Figure 1B). We further evaluated the scores of SRGs and discovered that T cells exhibited higher scores. This may suggest that abnormalities in sphingolipid metabolism within T cells are particularly pronounced in the ovarian cancer tumour microenvironment. High SRGs scores could potentially disrupt antitumour immune responses by modulating T cell activity 16 (Figure 1A-D). Interestingly, the activity of SRGs in ascites was significantly increased (*P*<2.2e-16), indicating that we can more sensitively detect the level of sphingolipid metabolism by collecting ascites from ovarian cancer patients (Figure 1E).

### Weighted co-expression network analysis (WGCNA)

**Figure 2:**
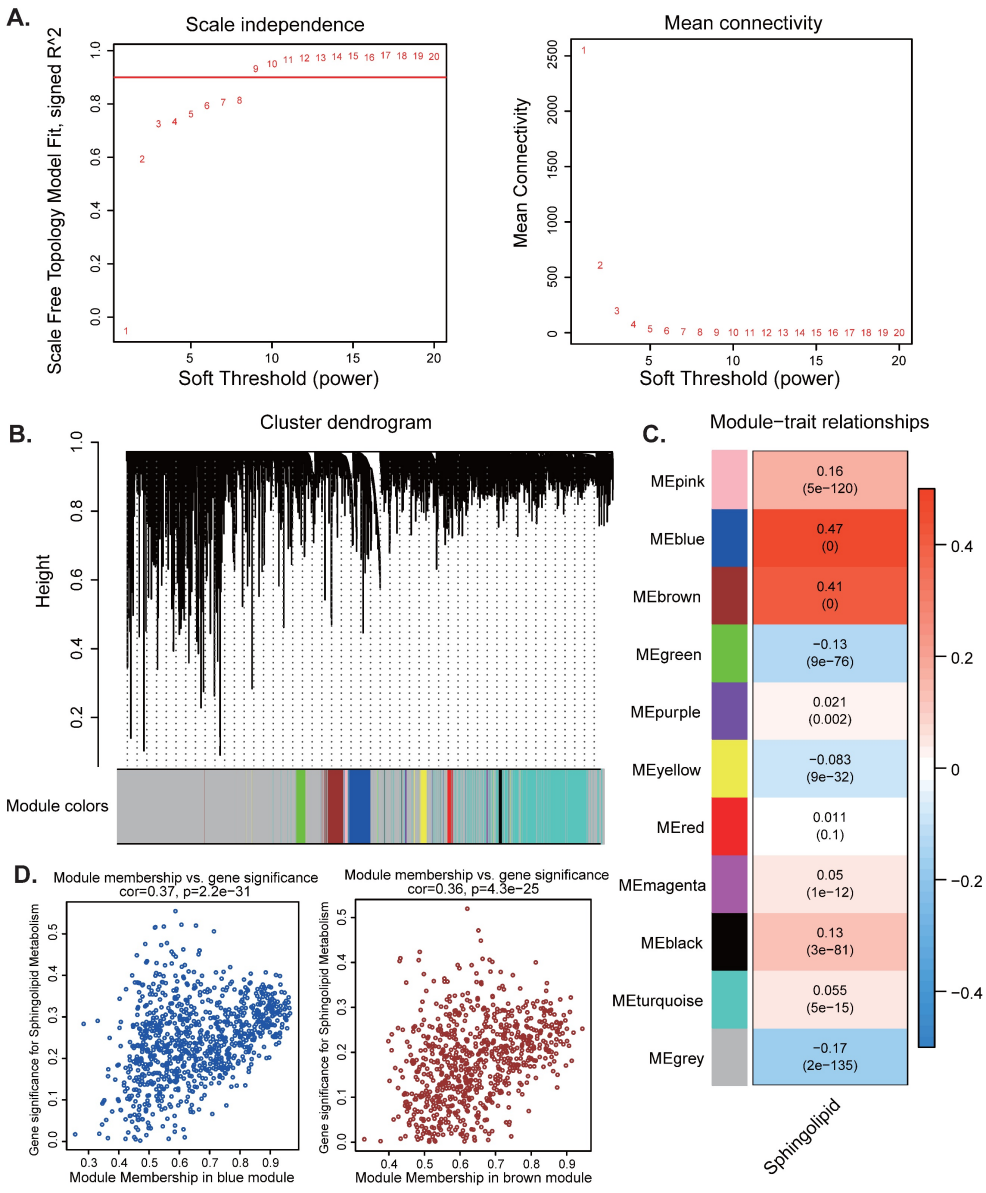
WGCNA analysis. (A-D) Weighted co-expression network analysis searched for the modules most associated with SM activity.

Subsequently, we aim to uncover potential gene modules that are significantly linked to sphingolipid metabolism. By leveraging the power of WGCNA, we can explore the intricate reltionships between genes and their regulatory networks, which may provide insights into the biological processes underlying sphingolipid metabolism. When the soft threshold value is set to 9, the data demonstrates heterogeneity in relation to power-law distribution, with the average connectivity tending to stabilize (Figure 2A). After merging modules with similarity below 0.25 and setting the minimum number of modules to 100 and deepSplit to 2, 12 non-gray modules were generated (Figure 2B). Among them, MEblue (COR=0.37, *P*<2.2e-31) and MEbrown module (COR=0.36, *P*<4.3e-25) are closely related to SRGs, among which MEblue contains 922 genes and MEbrown contains 777 genes are correlated with SRGs (Figure 2C-D). Therefore, we selected a total of 1699 genes from the two modules mentioned above for further analysis Table S1.

### Construction and validation of sphingolipid related prognostic model

**Figure 3:**
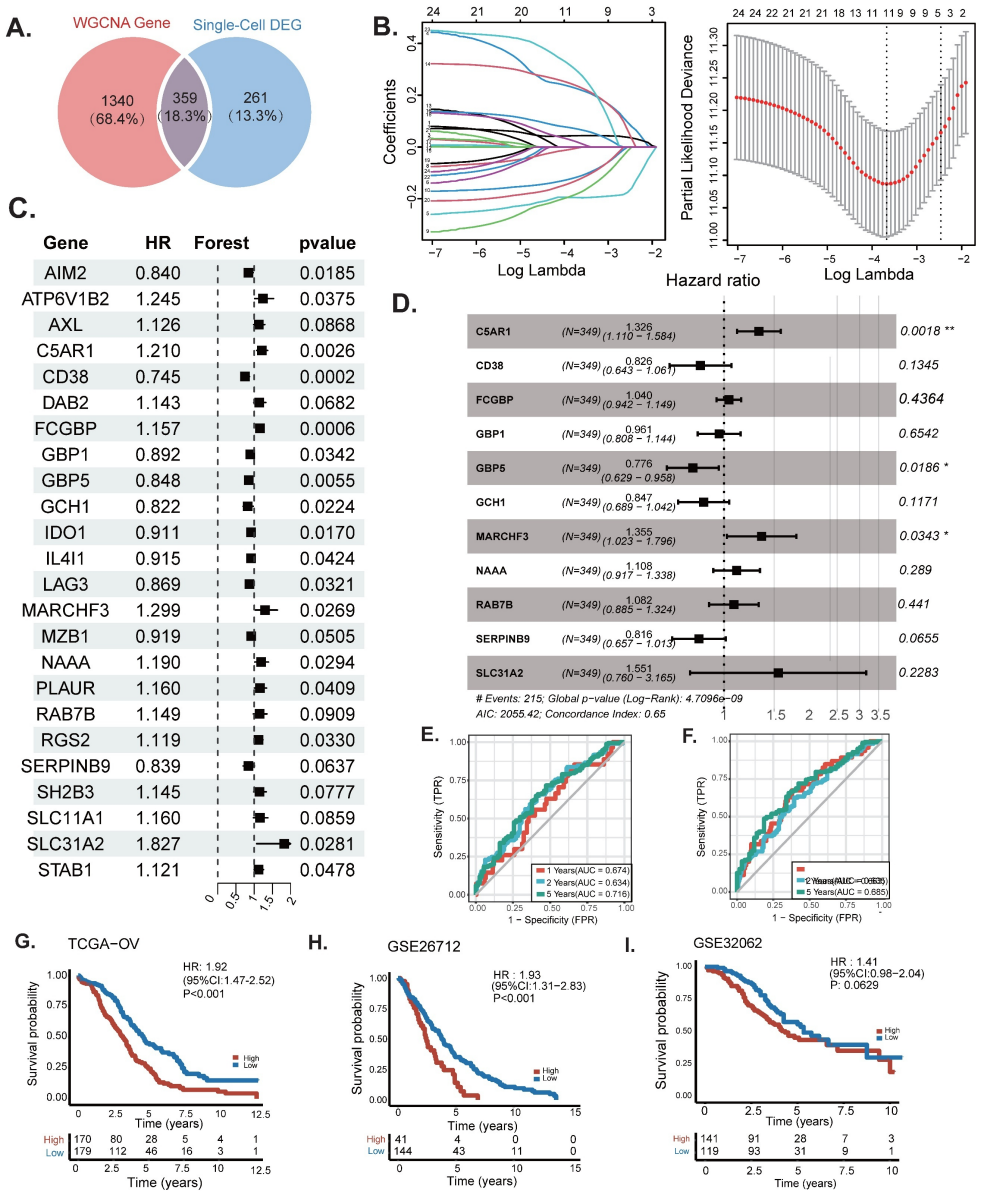
Construction and validation of sphingolipid-related prognostic model. The intersection of genes obtained in single-cell analysis and bulk-RNA analysis. (B)Genes significantly associated with prognosis after univariate regression. (C, D) Model genes and coefficients identified by Lasso regression and multivariate analysis. (E) Kaplan-Meier prognostic analysis of signatures in the TCGA-OV and GSE26712 cohorts. (F) The ROC curve was employed to assess the performance of the model in the training cohorts TCGA-OV, GSE26712, GSE32062.

To further explore the relationship between SRGs and the prognosis of OC patients, differentially expressed genes (calculated by categorizing tumor cells into high-expression and low-expression SRG groups, with genes defined as upregulated if p < 0.01 and Log2FoldChange > 0, totaling 620 genes) identified through single-cell analysis were intersected with the 1,340 genes most strongly associated with sphingolipid metabolism activity obtained from WGCNA. Ultimately, 359 overlapping genes were selected for subsequent analysis (Figure 3A) Table S1-3). A univariate Cox regression analysis was performed to identify prognostic genes linked to sphingolipid metabolism. Subsequently, LASSO regression analysis was utilised for dimensionality reduction (Figure 3B-C). A prognostic risk evaluation model based solely on 11 SRGs was constructed using the optimal penalty parameter (λ) derived from the aforementioned 24 SRGs in the training group. A multivariate Cox regression analysis identified three genes for model construction: C5AR1, GBP5, and MARCHF3 (Fig. 3D). We summarized the currently available studies in OC for the three differential genes mentioned above (Table 2. The study used the 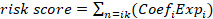 formula to construct a prognostic model, Coefi and Expi represented the coefficient and expression of each model gene, respectively, and the risk score for each sample was calculated by the above formula. Calculate the risk score of sphingolipid metabolism for each patient based on the expression of three genes, and divide them into high and low risk groups according to the median. Of the three genes used to construct the model, two were risk factors and one was protective factors (Figure 3D). In the TCGA-OV and GSE26712 cohorts, Kaplan Meier survival curves showed that patients in the high-risk group had significantly poorer prognosis compared to the low-risk group (*P*<0.001) (Figure 3G-H). Unfortunately, there was no significant difference in overall survival between the two groups in the GSE32062 cohort (*P*=0.0629) (Figure 3I). Furthermore, the ROC curves indicate that this model demonstrates commendable AUC values in both cohorts, with the TCGA-OV cohort recording AUCs of 0.674 at 1 year, 0.634 at 2 years, and 0.716 at 5 years (Figure 3G). In the GSE26712 cohort, the AUCs are 0.663 at 1 year, 0.635 at 2 years, and 0.685 at 5 years (Figure 3G). These findings suggest that the sphingolipid-related prognostic model possesses impressive accuracy in predicting the outcomes for ovarian cancer patients.

### Diagnostic nomogram model establishment

**Figure 4:**
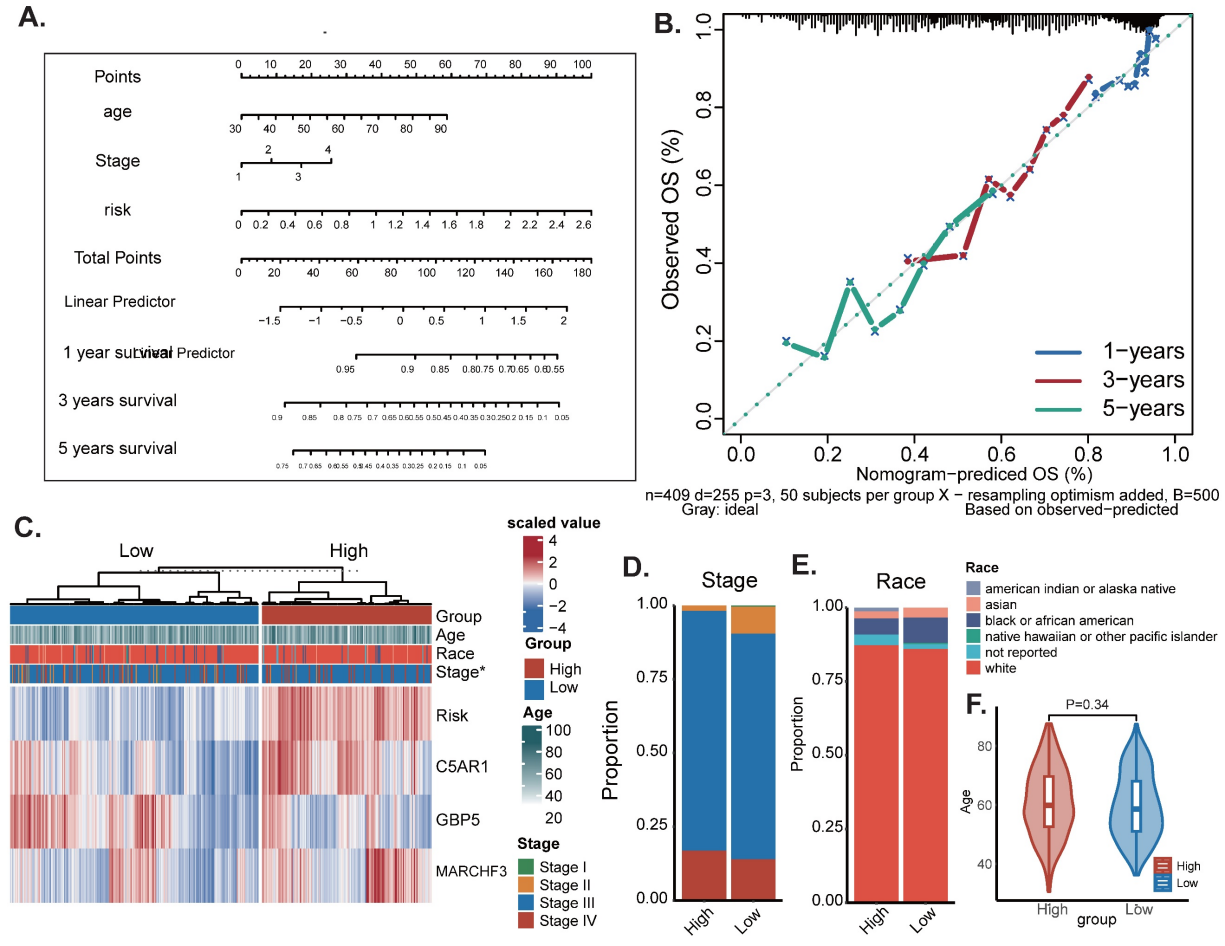
The Construction of a nomogram. (A) Nomogram to assess the risk of OC patients. (B) Calibration curves for 3- and 5-year specific survival rates. (C) Heat map incorporating clinical data, model genes. (D-F) The proportion of multiple clinical features between high-risk and low-risk groups: * *P* < 0.05.

To assess the potential clinical utility of prognostic features, a nomogram was developed based on the patients' clinical characteristics (including age and FIGO stage) as well as risk factors (Figure 4A). This nomogram could help more accurately determine the risk for ovarian cancer patients, ultimately providing valuable insights for future treatment decisions and improvements. The nomogram can help determine patient risk more accurately and direct future treatment decisions. The calibration plot is used to testify that the nomogram is consistent with our prediction, which showed good agreement with the actual outcome (Figure 4B). A heatmap was also generated showing the correlation between risk score prognostic features and clinical disease characteristics. According to the heatmap, the risk score presented is positively correlated with clinical staging (*P*<0.05, Figure 4B), while no statistical differences were found between other clinical features such as age and race. Then compare the distribution of various clinical features in each group and display it in a bar chart (Figure 4D-F).

### Mutation landscape analysis

**Figure 5:**
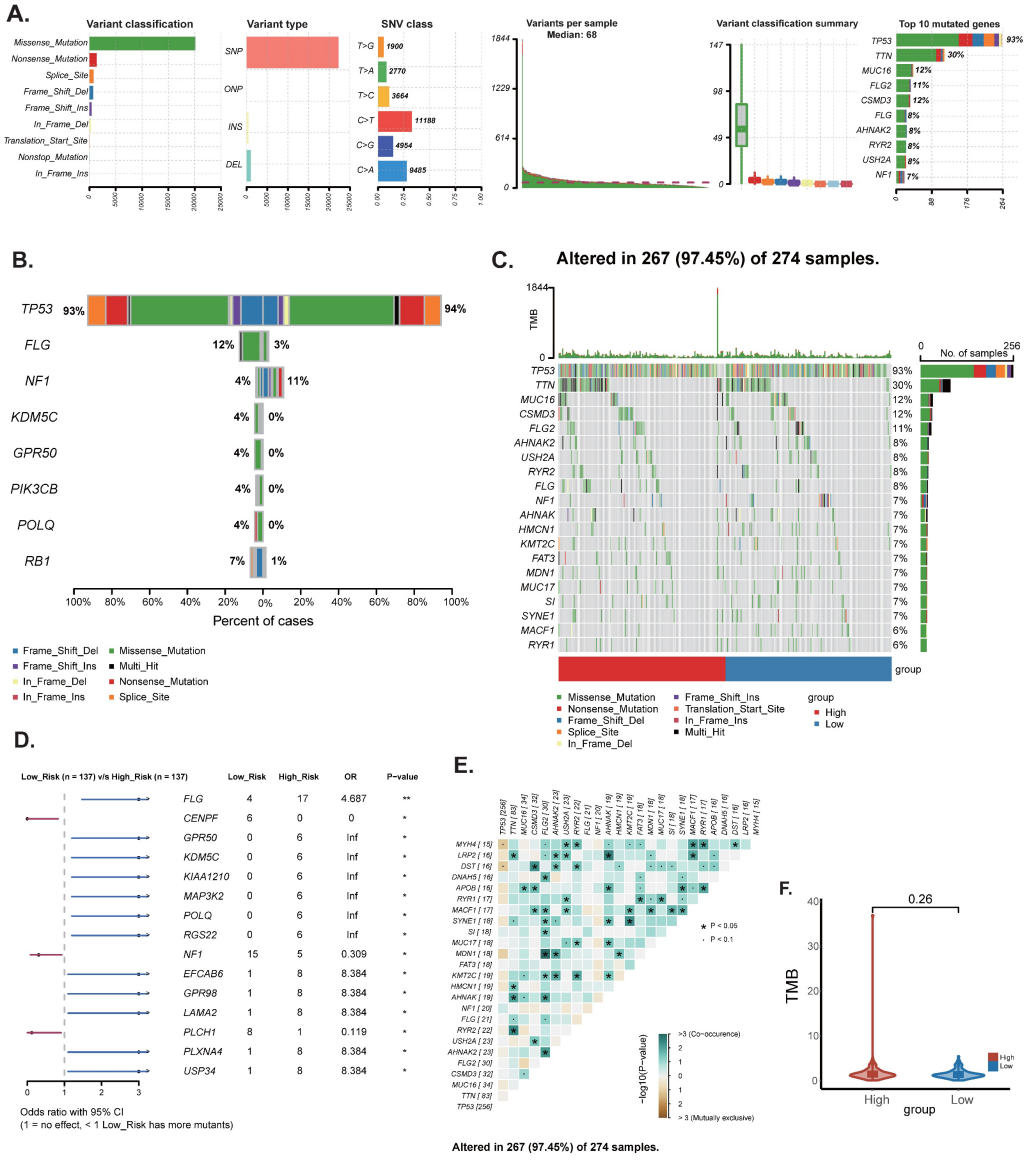
Landscape of OC sample mutation profiles. (A) Description of the statistical measurement mutation details, among which the most common mutation type was a missense mutation. SNP occupied an absolute proportion compared with insertion or deletion, and C>T occurred more frequently than in other classifications of forms. (B-C) Comparison of mutated genes and mutation landscape between high and low risk groups. (D) Forest map of mutated genes in different risk groups. (E) Co-mutation or co-exclusion relationships among model genes of the top 25 genes. (F) Comparison of TMB between different risk groups.

To determine the differences in cancer-related gene mutations between high-risk and low-risk groups, we counted the gene mutations in each group. The general information of two representative gene mutations is shown in Figure 5A, among which the most common mutation type was a missense mutation. The top 3 most frequent mutant genes were TP53, TTN, and MUC16 (Figure 5A). We also examined representative gene variants in the groups at high and low risk (Figure 5B) and further plotted the mutation landscape (Figure 5C). Genessuch as FLG, EFCAB6, GPR98, LAMA2, PLXNA4 and USP34 had the top six mutation frequencies in the high-risk group. The top three genes with the highest mutation frequencies in the low-risk group were CENPF, NF1 and PLCH1 respectively. Furthermore, we examined the mutation symbiosis of the top 25 genes and discovered that PIK3CA and NEB, MAP3K1, KMT2C, GATA3, CDH1, and TP53 all shared a mutation symbiosis (*P*<0.05, Figure 5C-E). Unfortunately, there was no significant difference in tumor mutation burden (TMB) between the high and low-risk groups (*P*=0.25) (Figure 5F).

### Pathway enrichment analysis

**Figure 6:**
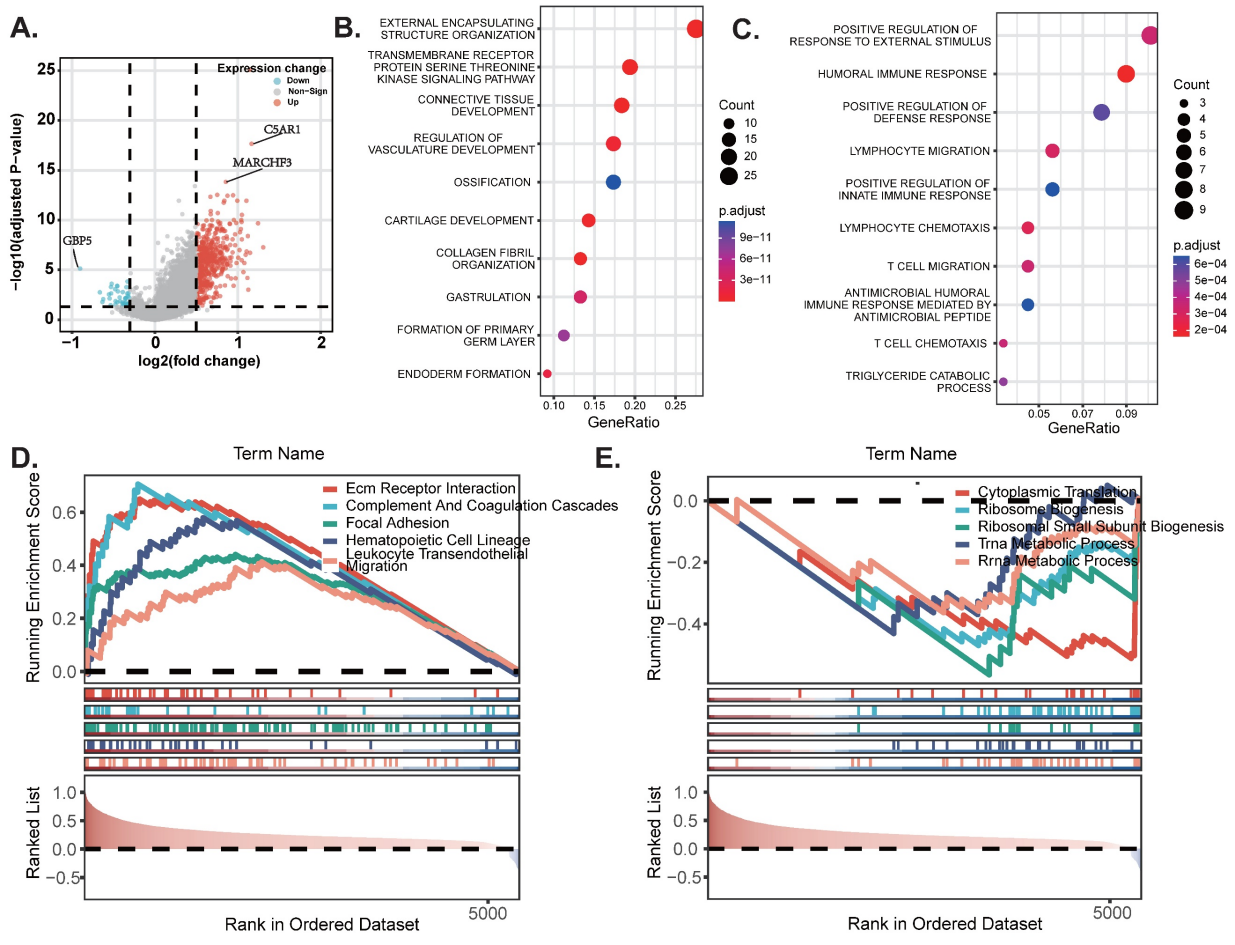
DEGs and pathway enrichment analysis. (A) Volcanic diagram of differentially expressed genes related to sphingolipid metabolism. (B-C) KEGG enrichment analysis of SRGs high and SRGs low. (D-E) GSEA studies of the biological processes and pathways enriched in SRGs high and SRGs low populations.

To begin with, we constructed a volcano plot to delineate the differential genes between the high-risk and low-risk groups (Figure 6A) Table S2. Furthermore, we conducted KEGG enrichment analysis on the differential genes from each group to investigate the potential functions of sphingolipid metabolism-related genes. The findings revealed that SRGs-high may be implicated in the regulation of transmembrane receptor protein serine/threonine kinase signaling pathways and the development of the vascular system, whereas SRGs-low appears to be associated with the positive regulation of fluid immune responses, T cell migration and chemotaxis (Figure 6B-C). In addition, we performed GSEA analysis to identify the most significantly enriched pathways distinguishing the two groups. Our analysis indicated that genes within the high-risk group were markedly enriched in extracellular matrix-receptor interactions and the complement and coagulation cascades signaling pathways (Figure 6D). Conversely, genes in the low-risk group exhibited significant enrichment in cytoplasmic translation and the biogenesis of ribosomal small subunits (Figure 6E).

### Immune landscape and analysis of drug sensitivity

**Figure 7:**
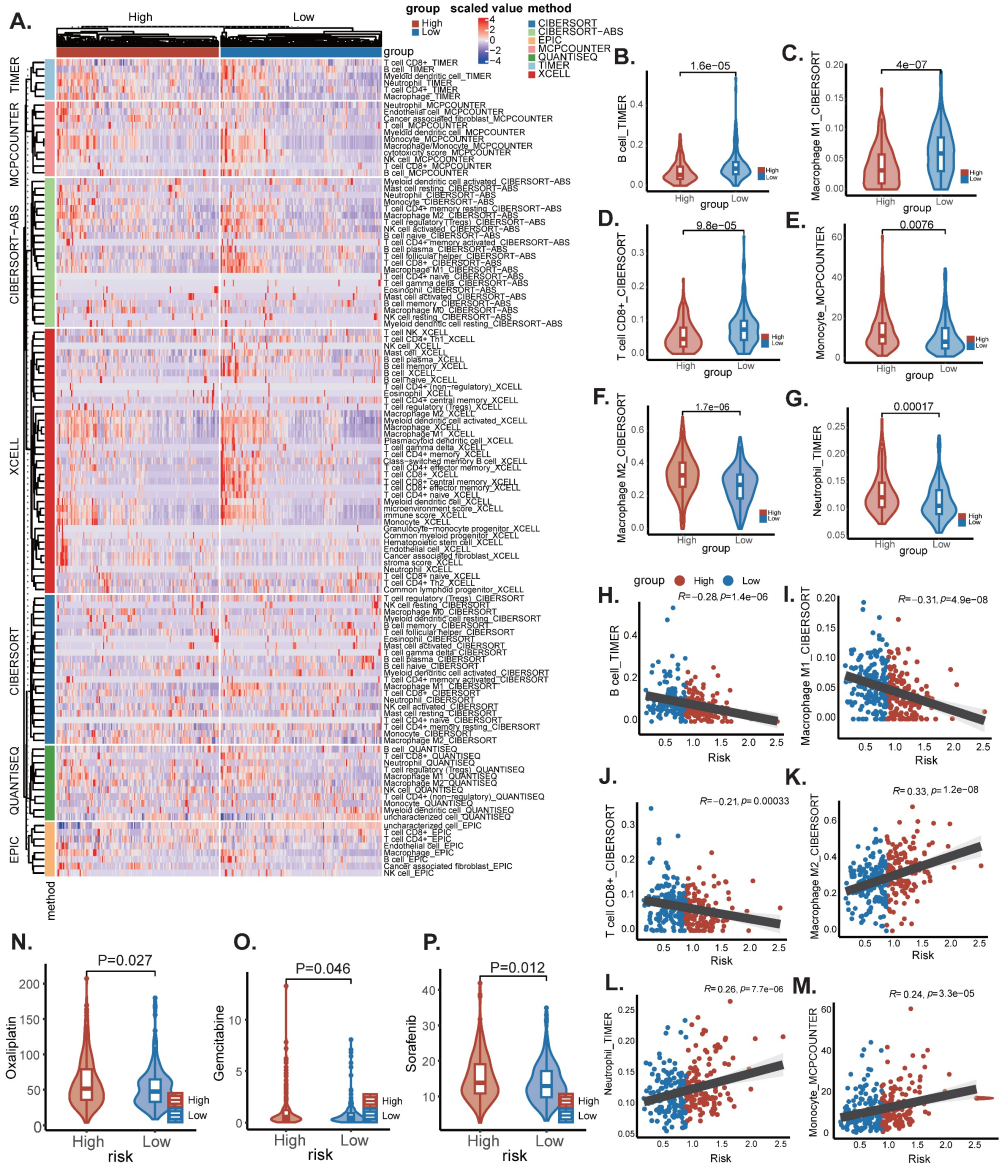
Immune infiltration and analysis of drug sensitivity. (A) There were significant differences in N stage, T stage, total stage, and survival between high and low-risk groups. (B) The age difference of patients between high and low-risk groups. (C) M stage difference of patients between high and low-risk groups. (D) N stage difference of patients between high and low-risk groups. (E) Total stage difference of patients between high and low-risk groups. (F-I) Potential drug screening in high-risk patients.

**Figure 8:**
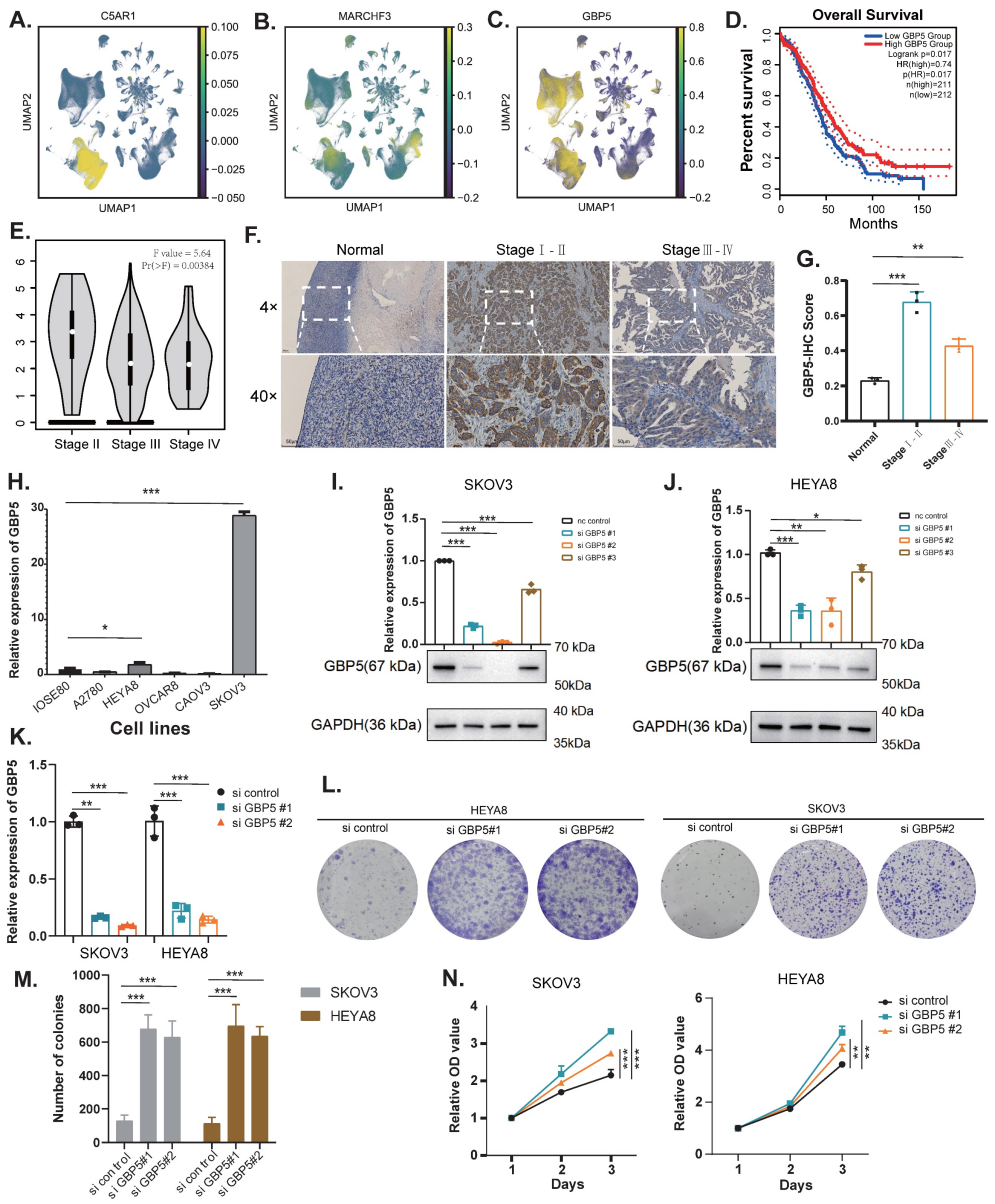
The expression of GBP5 in tissue samples and the verification of its knockdown in OC cell lines. (A-C) UMAP of key genes (C5AR1, MARCHF3, GBP5) with molecular characteristics related to sphingolipid metabolism in ovarian cancer. (D) Exploring the prognostic value of GBP5 expression in ovarian cancer using the GEPIA database (http://gepia.cancer-pku.cn/). (E) Exploring the value of GBP5 expression in clinical staging of ovarian cancer using GEPIA database (http://gepia.cancer-pku.cn/). (F-G) Immunohistochemistry (IHC) stain of GBP5 in clinical samples. (H) Expression of GBP5 gene in ovarian cancer cell lines. (I-K) Verification of the Knockdown Effect of GBP5 in SKOV3 and HEYA8 cell lines. (L-M) Cloning experiments after knocking down GBP5 in SKOV3 and HEYA8 cell lines. (N) CCK8 proliferation experiment after knocking down GBP5 in SKOV3 and HEYA8 cell lines (*P<0.05, **P< 0.01, ***P<0.001).

Tumor growth is severely affected by the tumor microenvironment, and due to the complexity of the immune microenvironment, OC is classified as a "cold tumor". Exploring the immune microenvironment landscape of high-risk and low-risk patients with SRGs may reveal potential mechanisms underlying survival differences between the two groups of patients. We used the CIBERSORT R script and analyzed using seven different algorithms to determine the degree of immune cell infiltration in each sample (Figure 7A). The results showed that the low-risk group tended to have increased infiltration of B cells, M1 macrophages, and CD8(+) T cells, compared to the high-risk group, which had increased infiltration of monocytes, M2 macrophages, and neutrophils (Figure 7B-M). Previous studies have shown that M2 macrophages accelerate the progression and metastasis of OC, while in contrast, high infiltration of M0 and M1 macrophages is associated with improved prognosis and therapeutic efficacy 17. In addition, Due to changes in certain gene levels of sphingolipid metabolism that may affect the effectiveness of chemotherapy, we further compared the differences in chemotherapy drug sensitivity between the two groups. We found that the IC50 values of Oxaliplatin, Gemcitabine, and Sorafenib (Figure 8N-P) were lower in the SRGs low group of patients, and these drugs may be candidate drugs for treating low-risk populations in clinical practice.

### The expression of key genes and the effect of knocking out GBP5 on cell proliferation

The UMAP plots illustrate the distribution of two genes, C5AR1 and MARCHF3, in the high sphingolipid metabolism scoring group, as well as the gene GBP5 in the low scoring group (Figure 8A-C). Referring to the cell subtype annotation results in Fig. 1B, C5AR1 exhibits a significant increase in expression within monocytes, while MARCHF3 is predominantly expressed in monocytes, fibroblasts, and T cells. Notably, GBP5 shows markedly elevated expression in both monocytes and T cells. Our attention was particularly drawn to GBP5, prompting us to utilise an online database (http://gepia.cancer-pku.cn/) to explore the correlation between GBP5 expression levels and survival outcomes in OC patients. The results indicated that patients with high GBP5 expression experience significantly prolonged OS (Figure 8D).

Subsequently, we found that early-stage (Stage I-II) OC patients exhibited significantly higher GBP5 expression levels compared to those with late-stage (Stage III-IV) ovarian cancer. Moreover, early-stage patients also demonstrated superior immunohistochemical scores relative to their late-stage counterparts (Figure 8E-G). We assessed GBP5 expression across several ovarian cancer cell lines compared to the normal ovarian epithelial cell line IOSE80 using RT-qPCR, revealing that GBP5 is relatively upregulated in the SKOV3 and HEYA8 cell lines (Figure 8H). Consequently, we conducted gene knockout of GBP5 using siRNA in these two cell lines. When compared to the si control group, GBP5 levels in SKOV3 and HEYA8 cells transfected with si GBP5#1 and si GBP5#2 were significantly suppressed at both the mRNA and protein levels (*P*<0.05) (Figure 8I-K). To further elucidate the impact of GBP5 on ovarian cancer phenotypes, we evaluated cell viability using the CCK8 assay. The results indicated that the absorbance values of GBP5 knockdown cell lines were higher than those of the control group. In contrast, the absorbance of the si-GBP5 transfected cell lines exceeded that of the control group (Figure 8A, 8E). In colony formation assays, both GBP5 knockdown cell lines demonstrated an increase in the number and volume of colonies formed compared to the control group (*P*<0.05), suggesting that GBP5 plays a role in suppressing ovarian cancer cell proliferation (Figure 8L-N).

### Knockdown of GBP5 promotes migration and leads to chemotherapy resistance

**Figure 9:**
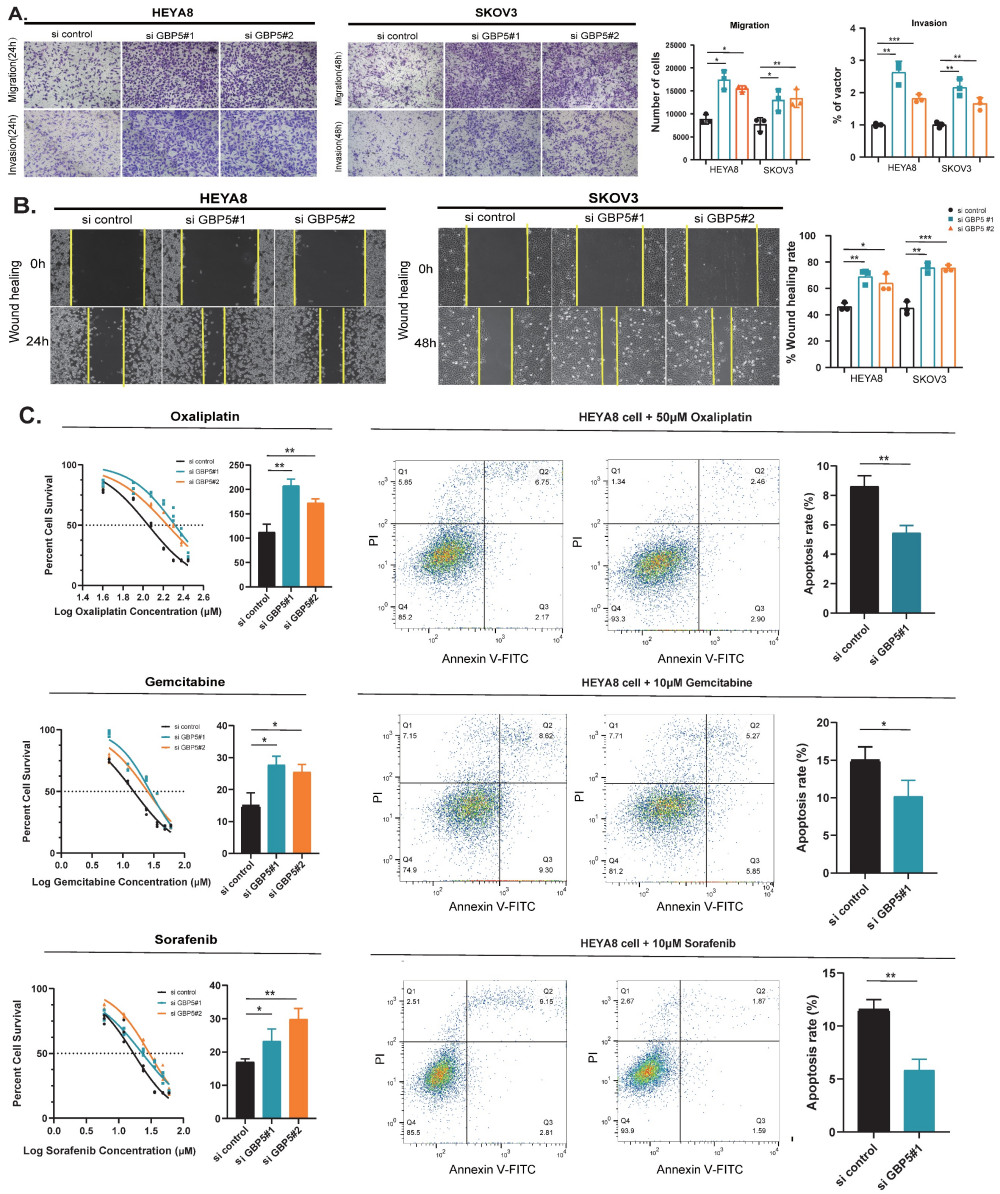
The effect of GBP5 knockdown on OC cell migration and sensitivity to chemotherapeutic drugs. (A) Transwell migration and invasion assays of si-GBP5 and control cells, with HEY cells observed for 12 hours and SKOV3 cells for 24 hours. (B) Wound healing assay of si-GBP5 and control cells, with HEY cells observed for 24 hours and SKOV3 cells for 48 hours. (C) Flow cytometry analysis of the effect of GBP5 knockdown on the percentage of HEYA8 cell apoptosis induced by oxaliplatin (50 μM, 48 h), gemcitabine (10 μM, 48 h), and sorafenib (10 μM, 48 h). The data shown represent mean ± standard deviation (*P<0.05, **P< 0.01, ***P<0.001).

Firstly, to ascertain the effect of GBP5 on the invasion and migration abilities of OC cell lines, Transwell assays revealed that the motility of GBP5-downregulated cells was significantly greater than that of the control group (Figure 9A). Similarly, the wound healing assay, designed to evaluate the migration capacity of OC cells, demonstrated that the healing rate of GBP5-downregulated cells was markedly higher than that of the control group (Figure 9B). In summary, these findings suggest that GBP5 knockdown promotes cell invasion and migration *in vitro*. Furthermore, to further investigate the impact of GBP5 on sensitivity to chemotherapeutic agents, we selected three drugs from Figure 7N-P that exhibited a significant reduction in IC50 in the SRGs-low group compared to the SRGs-high group: Oxaliplatin, Gemcitabine, and Sorafenib. After exposing HEYA8 cells transfected with GBP5 knockdown fragments and control fragments to various drug concentrations for 48 hours, we employed the CCK-8 assay to measure absorbance at 450 nm. As the drug concentrations increased, both groups displayed an elevation in proliferation inhibition rates. Notably, the IC50 of the GBP5 siRNA-transfected group was significantly higher compared to the control group (*P*<0.05). The IC50 values for the three drugs in the HEYA8 cell line were calculated as follows: for oxaliplatin treatment, the si GBP5#1 transfected group (207.47 ± 13.40) and si GBP5#2 transfected group (172.37 ± 8.06) were significantly higher than the control group (112.43 ± 16.25). For gemcitabine treatment, the values for the transfected and control groups were (27.75 ± 2.65), (25.47 ± 2.41), and (14.90 ± 4.08), respectively. Similarly, for sorafenib treatment, the control group showed a value of (16.94 ± 1.00), while the knockdown groups exhibited (23.22 ± 3.70) and (29.82 ± 3.29), indicating statistically significant differences (*P<0.05, **P<0.01, ***P<0.001). After 48 hours of induction with three drugs, flow cytometry analysis showed that knocking down GBP5 reduced the percentage of apoptotic cells in the HEYA8 ovarian cancer cell line (Figure 9C-E).

## Discussion

Ovarian cancer is the deadliest gynaecological malignancy, marked by significant tumour heterogeneity, a poor prognosis, and high mortality 18. At present, there are no reliable biomarkers to predict patient outcomes or assess treatment response effectively. Sphingolipids play a vital role as components of cell membranes and signalling molecules, influencing a range of biological processes, including apoptosis, proliferation, ageing, and stress responses 19. The involvement of sphingolipid metabolism in regulating the biological behaviour and heterogeneity of tumour cells is well established 20. Comparative studies of OC tissues against normal tissues have revealed numerous gene expression changes within the sphingolipid pathway. Ovarian cancer cells undergo metabolic reprogramming, resulting in an increased production of sphingolipid metabolism-related components, which alters their microenvironment and affects their sensitivity to chemotherapy drugs 21. These findings suggest that sphingolipid metabolism is a promising area of research in ovarian cancer therapeutics. Identifying specific sphingolipid metabolism-related targets and exploring the potential mechanisms across different tumour types is crucial. The aim of our study is to integrate the research linking sphingolipid-related genes with ovarian cancer prognosis, examine their potential clinical relevance, and provide new theoretical and practical frameworks for personalised treatment approaches.

In our study, we identified three hub genes: two risk hub genes, C5AR1 and MARCHF, and one protective hub gene, GBP5. C5aR1 and its ligand C5a are pivotal molecules within the complement system, and their interaction has been shown to regulate tumor growth and immune suppression in various cancers 22,23. Studies have revealed that C5a, within the tumor, binds to its receptor C5aR1 on myeloid-derived suppressor cells (MDSCs), which, through the recruitment of MDSCs into the tumor microenvironment, further suppresses CD8+ and CD4+ T cell-mediated anti-tumor immune responses, thereby promoting tumor growth 24,25. In OC, it has been observed that C5aR1 independently predicts poor prognosis and correlates with the infiltration of immunosuppressive cells within the tumor microenvironment, characterized by increased infiltration of pro-tumor cells (Treg cells, M2 polarized macrophages, and neutrophils) and impaired CD8+ T cell function, leading to poor responses to immune checkpoint inhibitors (ICIs) 26. Identification of C5aR1+ on tumor-associated macrophages (TAMs) indicates polarization towards an immunosuppressive phenotype; blocking the C5a/C5aR axis restores TAM anti-tumor responses and activates cytotoxic T cells, alleviating tumor progression 27. These findings are consistent with the gene characteristics we identified. On one hand, C5aR1, as a marker gene for high-risk groups, indicates poor prognosis in OC patients when highly expressed; on the other hand, there is increased infiltration of suppressive immune cell phenotypes in high-risk groups.

Therefore, targeting C5aR1 holds clinical potential, and combination therapy of anti-PD-1 antibodies with C5aR1 inhibitors may be a promising personalized treatment strategy. MARCHF is a subfamily of ubiquitin ligases that has been shown to play a role in immune responses and transmembrane transport 28. In colorectal cancer, MARCHF inhibits the activation of the IL-6 signaling pathway by ubiquitinating and degrading the IL-6 receptor, thereby attenuating the development of colitis and inflammation-associated tumor progression 29. In hepatocellular carcinoma, MARCHF regulates anti-tumor immune responses by degrading PARP1 and activating the cCAS-STING pathway in dendritic cells (DCs) 30. Specifically, the degradation of PARP1 promotes immune activation in dendritic cells, stimulating the STING (stimulator of interferon genes) pathway, thereby enhancing T cell-mediated anti-tumor immune responses. This process provides new molecular mechanisms for immunotherapy in hepatocellular carcinoma and suggests that MARCHF could be a potential target for anti-tumor immunity 30. However, no studies on MARCHF have been conducted in ovarian cancer to date. Our research shows that MARCHF is one of the high-risk genes associated with poor prognosis in OC. In the future, we look forward to further studies exploring the role of MARCHF in OC and its impact on prognosis. GBP5 is an important regulatory factor in the immune system, especially in regulating antimicrobial immunity and inflammatory responses. High expression of GBP5 in tumor cells may affect the immune environment by promoting autophagy and modulating anti-tumor immune responses. Zou *et al.* discovered that GBP5 serves as a protective gene in OC. On one hand, it inhibits ovarian cancer proliferation and metastasis by inducing classical pyroptosis via the JAK2/STAT1 pathway; on the other hand, GBP5 reprograms macrophages in the tumor-suppressive microenvironment towards M1 polarization, and its high expression increases the expression of programmed cell death ligand 1 (PD-L1), making it a potential survival marker for tumor patients receiving anti-PD1 and anti-PD-L1 therapy 31. Similarly, in our model, GBP5 is a protective gene, with its upregulation observed in the tumor tissues of early-stage OC patients compared to late-stage patients. Knockdown of GBP5 increases the proliferation, migration, and invasion capabilities of HEYA8 and SKOV3 cells, decreases sensitivity to chemotherapeutic agents such as oxaliplatin, gemcitabine, and sorafenib, and reduces apoptosis after treatment with these chemotherapy drugs. Further preclinical and clinical studies are needed to explore the impact of GBP5 on OC function and its tumor microenvironment.

Our findings indicate that increased expression of the risk hub genes correlates with a higher risk of OC, while the protective hub gene is associated with a reduced risk. Our investigation began with an in-depth analysis of single-cell heterogeneity, followed by the identification of key genes related to sphingolipid metabolism, complemented by bulk RNA sequencing analysis and the development of prognostic models for these three key genes. We constructed a nomogram to predict patient prognosis in ovarian cancer. Additionally, our study explored the expression of associated genes across different cell types and sphingolipid metabolism scores, examining immune infiltration and sensitivity to chemoresistance. Notably, our results suggest that the low-risk group exhibits greater sensitivity to oxaliplatin, gemcitabine, and sorafenib. We observed that GBP5, the most significantly different gene in the low-risk group, holds significant implications for ovarian cancer.

It is noteworthy that our model revealed an intriguing observation: in ovarian cancer, the high-risk group stratified by SRGs (sphingolipid-related genes) exhibited a strong correlation with an immunosuppressive tumour microenvironment characterised by elevated M2-polarised TAMs and reduced CD8+ T cell infiltration. A parallel phenomenon was observed in malignant melanoma by Mrad *et al.*, who demonstrated that inhibition of SPHK1 (the key enzyme catalysing sphingosine conversion to S1P) decreased M2 macrophage prevalence while increasing M1 macrophage proportions within the tumour niche. This M1 dominance enhanced CD8+ T cell infiltration and activation through augmented IL-12/TNF-α secretion, ultimately manifesting as elevated IFN-γ production and intensified tumour cell apoptosis 32. Some studies have uncovered potential mechanisms behind this phenomenon. On one hand, S1P, by binding to the S1PR1 receptor on macrophage surfaces, activates STAT3 phosphorylation, inducing the expression of anti-inflammatory factors such as IL-10, Arg1, and TGF-β, thereby driving M2 polarization 33. On the other hand, S1P upregulates HIF-1α, enhancing glycolytic capacity and promoting metabolic adaptation in M2 macrophages 34. Additionally, in breast cancer, increased enzymatic conversion of ceramide to sphingosine has been shown to reinforce M2 macrophage immunosuppression via PI3K-AKT-mTOR pathway activation 35. Collectively, these findings underscore that sphingolipid metabolism may establish an immunosuppressive niche through synergistic mechanisms. Overall, sphingolipid metabolism may establish an immunosuppressive niche through various synergistic mechanisms, and targeting specific nodes within this network is worth further investigation as part of combination immunotherapy strategies.

Overall, our study has contributed to a deeper understanding of the biological processes involved in sphingolipid metabolism in ovarian cancer, providing insights for diagnosis, treatment, and potential combination therapies. However, several limitations of this study warrant acknowledgment. Firstly, the origins of OC are diverse, and our research focused solely on epithelial-derived OC samples, making it challenging to generalise the model to other OC types. Secondly, due to the lack of available data, a critical clinical feature—TNM staging—was not incorporated into the prognostic model. TNM staging is a key factor in evaluating tumor progression and prognosis, and the absence of this data limits our ability to perform a more comprehensive analysis of the relationship between the studied biomarkers and clinical outcomes based on tumor staging. Furthermore, we did not conduct *in vitro* or *in vivo* functional studies on the high-risk central genes, which constrains our understanding of their precise mechanisms of action. Additionally, the relatively small sample size used in our study may affect the robustness and stability of our models. It is crucial to note that this study is primarily retrospective and did not utilise prospective data to test the model's performance. It is also important to note that this study was predominantly retrospective and did not utilise prospective data to test the performance of the model. This introduces potential biases, such as selection bias and reliance on existing medical records, which may not always be complete or consistently collected. Therefore, prospective studies with more controlled methodologies are necessary to confirm the observed associations and further explore their clinical relevance. Larger prospective datasets are required to validate the features identified in this study and enhance their accuracy and reliability. Future research could also delve into the specific characteristics and genetic features of these subtypes, contributing to a more comprehensive understanding of ovarian cancer heterogeneity in its broader context and extending applicability to different OC types.

## Conclusion

Conclusively, we developed and validated a novel prognostic signature comprising of 3 SRGs marker genes by integrated analysis of single-cell and bulk RNA-sequencing, which outweighs other well-established signatures in predicting the prognosis of OC patients and might predict patients' response to chemotherapy in OC. Our analysis of indicated their potential roles in the prognosis value and tumor immune microenvironment. Furthermore, we have verified the function of GBP5 in OC through cellular experiments and its impact on chemotherapy sensitivity after altering GBP5 expression in ovarian cancer cells. These results might offer useful information for creating fresh OC treatment plans.

## Supplementary Material

Supplementary tables.

## Figures and Tables

**Table 1:** siRNA sequences.

siRNA ID	Sense Strand (5'→3')	Antisense Strand (3'→5')
si NC	UUCUCCGAACGUGUCACGU	GUGACACGUUCGGAGAATT
si GBP5#1	GCAGCACCUUUGUGUACAA	GCAGCACCUUUGUGUACAA
si GBP5#2	CUAUCGACCUACUGCACAA	UUGUGCAGUAGGUCGAUAG
si GBP5#3	GGUCAAUGGAUCUCGUCUA	UAGACGAGAUCCAUUGACC

**Table 2:** The discovered functions and prognostic value of three differentially expressed SRG related genes in ovarian cancer.

Gene	Gene name	Functions in OC	Reference
C5AR1	Complement Component 5 Receptor 1	High expression indicates poor prognosis and facilitates the infiltration of Tregs and M2 macrophages, leading to the formation of an immunosuppressive tumor microenvironment.	(26,27)
MARCHF3	Membrane-Associated RING Finger Protein 3	None	None
GBP5	Guanine Nucleotide-Binding Protein 5	Inducing cell pyroptosis through the JAK2-STAT1 signaling pathway and enhancing polarization of M1 macrophages in the tumor microenvironment.	(31)

## Abbreviations

SRGs: sphingolipid-related genes
OC: ovarian cancer
scRNA-seq: single-cell sequencing analysis
LASSO: least absolute shrinkage and selection operator
EOC: epithelial ovarian cancer
Cers: ceramides
SMs: sphingomyoelins
Sph: sphingosine
S1P: sphingosine 1-phase
PD-L1: programmed death ligand 1
HGSOC: high-grade serous ovarian cancer
OS: overall survival
UMI: unique molecular identifier
PCA: principal component analysis
UMAP: uniform manifold approximation and projection
DCs: dendritic cells
WGCNA: weighted gene co-expression network analysis
DCA: decision curve analysis
TME: tumor microenvironment
ssGSEA: single-sample Gene Set Enrichment Analysis
IC50: inhibitory concentrations
IHC: immunohistochemistry
RT-qPCR: reverse transcription quantitative PCR assay
CCK-8: cell counting Kit-8
SD: standard deviation
TMB: tumor mutation burden
