# Supplementary material for: Integrating Single-Cell and Bulk RNA Sequencing Data to Explore Sphingolipid Metabolism Molecular Signatures in Ovarian Cancer Prognosis: an Original Study
Source: Int J Med Sci. 2025 Mar 24;22(8):1958–77. doi: 10.7150/ijms.107391 (PMC11983299; doi:10.7150/ijms.107391)
Supplement: Supplementary file 1 — Supplementary tables. [file ijmsv22p1958s1.zip › Supplementary materials/Supplementary materials.pdf]

Table S1. List of genes identified by WGCNA. Using WGCNA to depict gene modules associated with SRG scores in OC and identify the genes contained within the MEbrown and MEblue modules.

Table S2. List of differentially expressed genes in single-cell data. Identifying differentially expressed genes between high-risk and low-risk SRGs based on single-cell sequencing data.

Table S3. Differentially expressed genes (calculated by categorizing tumor cells into high-expression and low-expression SRG groups, with genes defined as upregulated if $p < 0.01$ and Log2FoldChange > 0, totaling 620 genes) identified through single-cell analysis were intersected with the 1,340 genes most strongly associated with sphingolipid metabolism activity obtained from WGCNA. Ultimately, 359 overlapping genes were selected for subsequent analysis.

Table S4. Studies on ovarian cancer cell lines related to sphingolipid metabolism.

Cell line	Characteristics	Researches on SMs
A2780	Platinum-sensitive, TP53 wild-type (baseline chemo-response model).	Accumulation of ceramide in A2780 cells enhances cisplatin-induced apoptosis (36).
OVCAR8	BRCA1/2-mutant (homologous recombination deficiency studies).	Ceramide analogues, such as C6-ceramide, induce autophagic cell death in OVCAR8 cells (37).
SKOV3	Platinum-resistant, EGFR-amplified (drug resistance studies).	Exosomes secreted by SKOV3 are enriched in S1P and, via paracrine signalling, suppress T cell activity (38).
HEYA8	Metastatic ascites-derived, MYC-driven (TME interaction modeling).	In HEYA8 cells, ceramide synthase 2 is upregulated under ascitic-like conditions, promoting invasion (36).
CAOV3	Platinum-resistant, TP53-mutant (drug resistance studies).	CAOV3 cells promote M2 polarization of TAM via the S1P/NF-κB axis (39).
